# Nanoparticles as Versatile Tools for Mechanotransduction in Tissues and Organoids

**DOI:** 10.3389/fbioe.2020.00240

**Published:** 2020-04-17

**Authors:** Abdel Rahman Abdel Fattah, Adrian Ranga

**Affiliations:** Laboratory of Bioengineering and Morphogenesis, Department of Mechanical Engineering, KU Leuven, Leuven, Belgium

**Keywords:** nanoparticles, organoid, hydrogel, tissue engineering, synthetic microenvironments

## Abstract

Organoids are 3D multicellular constructs that rely on self-organized cell differentiation, patterning and morphogenesis to recapitulate key features of the form and function of tissues and organs of interest. Dynamic changes in these systems are orchestrated by biochemical and mechanical microenvironments, which can be engineered and manipulated to probe their role in developmental and disease mechanisms. In particular, the *in vitro* investigation of mechanical cues has been the focus of recent research, where mechanical manipulations imparting local as well as large-scale mechanical stresses aim to mimic *in vivo* tissue deformations which occur through proliferation, folding, invagination, and elongation. However, current *in vitro* approaches largely impose homogeneous mechanical changes via a host matrix and lack the required positional and directional specificity to mimic the diversity of *in vivo* scenarios. Thus, while organoids exhibit limited aspects of *in vivo* morphogenetic events, how local forces are coordinated to enable large-scale changes in tissue architecture remains a difficult question to address using current techniques. Nanoparticles, through their efficient internalization by cells and dispersion through extracellular matrices, have the ability to provide local or global, as well as passive or active modulation of mechanical stresses on organoids and tissues. In this review, we explore how nanoparticles can be used to manipulate matrix and tissue mechanics, and highlight their potential as tools for fate regulation through mechanotransduction in multicellular model systems.

## Introduction

Over the last decades advances in methods to precisely direct stem cell fate have enabled the generation of increasingly biomimetic models of human development in a dish, and the translation of these approaches to disease-specific models has led to important insights into the etiology of pathological states. Stem cell-derived organoids have enhanced our ability to mimic human physiology by providing multicellular, tissue-like organization, allowing for modeling of complex tissue functions and disease phenotypes. In particular, human induced pluripotent stem cell (iPSC)-derived organoids begin to recapitulate key features of human-specific developmental steps and pathological features which are impossible to mimic with animal models (Lancaster and Huch, 2019). The *in vitro* aspect of organoid culture, and the use of biomaterials to rationally design their surrounding microenvironment, provides the freedom to interrogate the specific role of biochemical and mechanical cues in determining cell fate specification, morphogenesis and patterning. In particular, mechanical stresses imparted by dynamic tissue deformation during development are increasingly recognized as critical sources of timed inductive cues with important regulatory roles. To sense these cues, cells rely on their ability to receive and process external changes in the biophysical environment to activate genetic programs driving specific responses, a process known as mechanotransduction (Chan et al., 2017; Davidson, 2017). The interpretation of mechanical signals is performed by specialized mechanosensitive and mechanotransductive proteins, whose dysregulation leads to important pathologies. Vinculin is one such critical element in mechanoregulation, serving to link integrins with the cytoskeleton, and vinculin mutations in mouse embryos are associated with severe neural tube defects (Xu et al., 1998).

**FIGURE 1:**
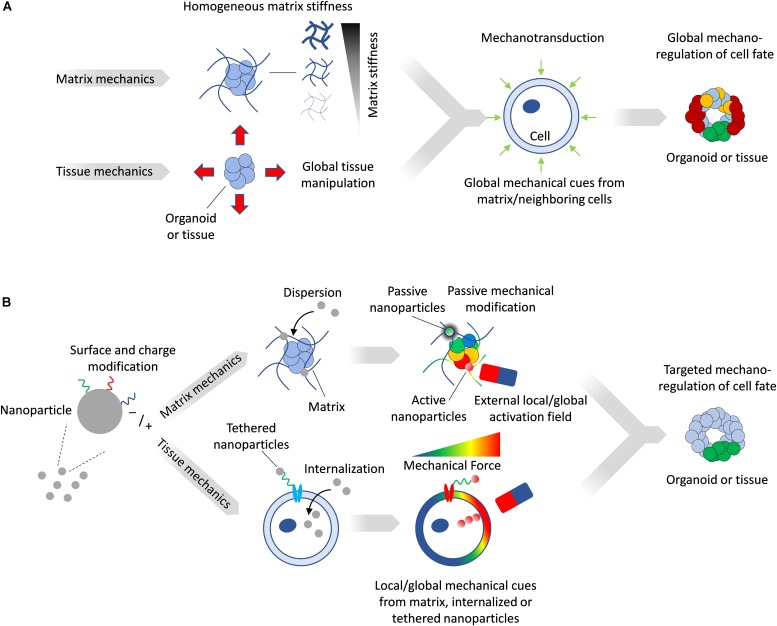
**(A)** Matrix mechanics can be modulated to create stiff or soft synthetic microenvironment, while device-driven tissue manipulation can impose mechanical stresses directly on tissues. Such manipulation provides global and homogenous mechanical cues on tissues and organoids. **(B)** Magnetic NPs can be tailored to matrix or tissue mechanics applications. NPs activated by a field can generate local mechanical forces, while inactive ones can passively alter local matrix stiffness. Magnetized cells can be subjected to forces by an external field and transfer these mechanical stresses to the surrounding tissue.

The *in vitro* modulation of the mechanical microenvironment can be broadly characterized as being either passive or active. Passive modulation consists of setting a mechanical milieu within which cells can interact but which cannot be changed, such as the culture of cells on or within an extracellular matrix (ECM) of specific stiffness. Landmark studies have established that substrate stiffness alone can direct stem cell fate, with, for example, mesenchymal stem cells adopting the fates of tissues whose stiffness corresponds to that of the substrates on which they were cultured (Engler et al., 2006). Active mechanical modulation of the microenvironment provides for controlled changes in the stress/strain fields, which can be actuated externally. In both cases, the entire multicellular construct is subject to a homogenous mechanical state (Figure 1A) which can be different from that of the *in vivo* scenario, where specific regions of the tissue may experience local and anisotropic mechanical stresses and deformations. To overcome these limitations, increasingly biomimetic *in vitro* technologies for modeling tissue mechanobiology are beginning to incorporate features of the complex and dynamic mechanical interplay between tissues. Here, we provide a brief overview on the role of mechanobiology in tissue development, review current methods to alter matrix and tissue mechanics *in vitro*, and illustrate how nanoparticles (NPs) provide additional design parameters offering unique capabilities to engineer the mechanical microenvironment in tissues and organoids with a high degree of spatial and temporal resolution.

## Role of Mechanical Cues in Developing Tissues

Continuous tissue deformations underlie the early development of all tissues, and are orchestrated by 3D cell rearrangements, sorting, migration and differentiation. The toolkit of morphogenesis includes mechanisms such as differential adhesion, epithelial-to-mesenchymal and mesenchymal-to-epithelial transitions, as well as apical constrictions and intercalations, which all contribute to the generation of intracellular mechanical forces. Classic *in vitro* cell reaggregation experiments have shown that cells of the same type can sort within a mixed population to give rise to an ordered construct (Steinberg, 1963), based on expression of junctional cadherins and catenins. Similarly, the minimization of free energy can help explain *in vitro* germ layers stratification based on differential surface tensions (Davis et al., 1997). Additionally, integrin-mediated interactions between cells and ECM have been shown to play a critical role in enabling large-scale tissue motions during convergent extension and somitogenesis (Bénazéraf et al., 2010). The ECM is also involved in regulating cavity and lumen formations in epithelial tissues throught its role in cytoskeletal modifications via integrin binding (Bedzhov and Zernicka-Goetz, 2014). At the scale of the single cell as well as at multicellular and tissue scales, these deformations contribute to the establishment of mechanical fields which feed back onto gene regulatory networks. The development of the embryonic gut provides an illustrative example of how differential growth rates at local scales lead to large-scale changes in morphology. Here, the, differences in growth rate between the gut tube and mesentery cause an initially straight structure to loop due to buckling forces along its long axis (Nerurkar et al., 2017).

The molecular mechanisms of mechanosensing and mechanotransduction include factors such as the Hippo pathway effector Yap (Lian et al., 2010; Dupont et al., 2011; Benham-Pyle et al., 2015) as well as the mechanically gated ion channels Piezo1/2 (Pathak et al., 2014; Hennes et al., 2019), which produce their effects either indirectly, e.g., through Yap nuclear translocation, or directly, e.g., through the nucleocytoskeleton and cytoskeleton (LINC) complex (Stewart et al., 2015). Indeed, Yap changes its localization from cytoplasmic (no mechanical stimulation/inactive form) to nuclear (under mechanical stimulation/active form) (Dupont et al., 2011) and Yap has been shown to play an important role in stem cell differentiation, with its levels gradually decreasing during the transition from pluripotency (Lian et al., 2010). Moreover, Yap, in concert with β-catenin, has been shown to initiate cell cycle reentry when active mechanical forces are present (Benham-Pyle et al., 2015). Independently of the Hippo pathway, the pressure-activated cation channels Piezo1 and Piezo2 have been shown to be direct sensors of fluid sheer stress and membrane stretch, converting applied force into electrical signals (Lin et al., 2019) in such varied contexts as vascular endothelium, pancreas and skin. Piezo1 has notably also been involved in determining lineage choice in human neural stem cells, where activation by traction forces resulted in neurogenesis, while inhibition gave rise to astrocytes and limited neuronal differentiation (Pathak et al., 2014). Another study in human endometrial epithelial cells (hEECs) demonstrated that activation of Piezo1 was a direct response to cell membrane mechanical indentation, suggesting such mechanoregulation may be important in embryo implantation (Hennes et al., 2019). Interestingly, endometrial organoids were used here as a model system which could sustain longer culture under mechanical activation compared to primary hEECs. These examples illustrate that while the elements of the cellular mechanosensing and mechanotransducing machinery are the same in various tissues and spatial configurations, they are highly context-specific in how they are deployed and in their role.

## Artificial Extracellular Matrices to Engineer Microenvironmental Control

Organoids provide tractable model systems to deconvolve the complex and multifactorial interplay between mechanics and biological response. As such, matrix engineering has provided a powerful avenue to controllably modulate the mechanical microenvironment *in vitro*. Naturally derived matrices such as collagen and Matrigel have routinely fulfilled the role of supportive host matrix required for the emergence of characteristic three-dimensional organoid features. However, these materials suffer from batch-to-batch variability and lack mechanical tunability, which hinders efforts to understand the role of individual microenvironmental elements. Artificial extracellular matrices (aECM) have emerged as important alternatives to overcome these limitations by mimicking specific elements of natural ECMs in a more controllable manner. Their mesh-like or fibrillar network composition in the form of hydrogels provides mechanical support, and such aECMs can be engineered to allow for remodeling capabilities, for example by cell-secreted matrix metalloproteinases (MMPs). The high degree of control over their mechanical characteristics through changes in the density of the polymer subunits has been employed to direct cell fates, with common examples including the control of embryonic stem cell pluripotency (Li et al., 2006) or the differentiation of human mesenchymal stem cells (hMSCs) to chondrogenic fates (Kloxin et al., 2009). As in natural ECMs such as laminin, fibronectin and collagen, aECMs can be engineered to present integrin-binding sites (e.g., RGD) which enable cell traction and migration. The modularity and orthogonality of these properties has made the multiplexing of the combinatorial experimental design space possible. This has allowed for high throughput screening of matrix characteristics such as stiffness, presence and concentration of adhesion ligands and degree of degradation by cellular proteolytic activity (Ranga et al., 2014). Such platforms have been used to uncover the role of the matrix in diverse contexts such as cytoskeleton-driven symmetry breaking in a mouse neural tube organoid model (Ranga et al., 2017) and in reprogramming of iPSCs (Caiazzo et al., 2016).

Matrices with dynamic mechanical characteristics, which can transition from stiff to soft (Gjorevski and Lutolf, 2017) or from fluid to solid (Bhattacharjee et al., 2015) in a defined manner can provide additional dynamic control. These can provide timed mechanical cues and respond to the transient mechanical needs of organoid culture. For example, PEG–based aECMs have been used to elucidate the role of dynamic mechanical forces in intestinal stem cell expansion and organoid formation (Gjorevski et al., 2016) by rendering gels degradable in a time-dependent manner though the incorporation of hydrolytically degradable moieties in an otherwise non-degradable gel. Stiff matrices were shown to create ideal conditions for intestinal organoid colony formation, but not necessarily for organoids morphogenesis, i.e., crypt budding. By rendering the matrix degradable only at a later time, the initially stiff matrices could support colony formation and, upon degradation, favored organoid growth. Such modulation, translated molecularly via Yap1 (Gjorevski and Lutolf, 2017), could not be achieved in a matrix with fixed mechanical characteristics, and is a striking example of the importance of tunable aECMs. Similar degradable hydrogels have shown promise for the growth and expansion of human intestinal organoids derived from iPSCs, as well as for the successful engraftment of organoids containing hydrogels in mice for colonic wound repair applications (Cruz-Acuña et al., 2017).

In addition to cell or hydrolysis-mediated mechanical modifications, other environmental conditions such as pH and temperature can be modulated exogenously to control matrix properties and resulting organoid characteristics. For example, glioblastoma-derived cells could be cultured as tumoroids to a specified size in thermoreversible PNIPAAM-PEG hydrogels, whereupon a rapid change in temperature to 4°C caused the matrix to liquefy and free the cells, making them available for further expansion (Li Q. et al., 2016). Compared to conventional methods, this approach yielded 20-fold higher cell density while limiting aggregation. For *in vivo* applications, pH sensitivity can be more beneficial than thermoresponsiveness, since slight changes in pH can occur between diseased and healthy tissues, offering a parameter for targeting the diseased region. For instance, cardiosphere-derived cells were embedded in hydrogels that only polymerize at a pH of 6.5 and at 37°C, resembling conditions in infarcted hearts (Li Z. et al., 2016).

A disadvantage of temperature and pH-sensitive matrices is that their property changes often involve phase changes which can disrupt cellular spatial organization. Light-sensitive photodegradable materials can provide post-gelation mechanical tuning *in situ* with more gradual property modifications. In one study, ultraviolet light has been used to reduce the stiffness of photodegradable hydrogels from 10 to 2 KPa, thereby allowing *in situ* manipulation of the mechanical cues perceived by MSCs (Yang et al., 2014). These cells we shown to retain memory of stiff microenvironments by committing to an osteogenic fate despite the substrate transition from stiff to soft, which would normally favor softer tissues. An additional advantage of photosensitive materials is that spatial control over properties can be achieved, *in situ* and post-gelation (Kloxin et al., 2009). In one example, hMSCs were embedded in hydrogels with photolabile groups and targeted gel photodegradation was performed by two-photon microscopy, forming 3D internal channels through which cell could undergo directed cell migration. Precise 3D control of photodegradation over mechanical stresses, as shown here, could open interesting avenues for investigating how precisely shaped stress fields could control organoid growth.

Optimizing matrix properties *in vitro* can also have an important *in vivo* role in therapeutic applications. For example, a PEG hydrogel with a stiffness of 350 Pa promoted differentiation of iPSCs to a cardiac fate, with the resulting hydrogel-cell bioactive tissue achieving a high degree of repair when injected in infarcted mouse hearts (Bearzi et al., 2014). Further optimization of such aECMs can also be targeted toward practical purposes such as improving material handling and delivery by enhancing injectability or by enabling on-site polymerization (Chow et al., 2017).

## Extrinsic Control of Cellular Forces Through Motorized Devices and Optical Tweezers

In addition to designing microenvironments with specified mechanical properties, active force modulation can also be imposed independently of matrix engineering, and can be beneficial to mimic the chronology of *in vivo* deformations. A common way to achieve this at multicellular resolution is through the use of instruments where cells are plated on elastomeric membranes which undergo mechanical stretching. By stretching the membranes, strains are transferred to the adhered cells, subjecting them to mechanical stresses. Such experiments help in understanding the role of tissue deformation in specifying cell fate in highly controlled settings, and can identify the mechanosensitive pathways involved. For example, cyclic loading of fibroblast cells have shown Zyxin to be an important mechanosensitive protein, playing a role in filamentous actin remodeling and reinforcement (Hoffman et al., 2012). In some applications, more elaborate devices are necessary to provide complex strain field modulation, such as in the case of a microfabricated pneumatic device used to provide cyclic loading via inflatable membranes to differentiating human pluripotent stem cells (hPSCs) (Xue et al., 2018). The mechanical stimulation of hPSCs, together with exogenous BMP signaling, was shown to alter neural plate border patterning, highlighting how biophysical parameters could synergize with biochemical signals to induce neuroectoderm patterning in 2D.

Other methods such as optical tweezers have been employed to directly impose pN forces at the length scale of the single cell. This is achieved by tethering polystyrene beads to cell membranes, which can be manipulated when subjected to focused light. Using this technique, a recent study highlighted how mouse neuroblastoma cells respond to forces ranging from 5 to 20 pN by activation of Ca^2+^ ion channels at defined force thresholds (Falleroni et al., 2018). Optical tweezers have been largely applied and optimized to 2D single cell scenarios and thus may prove less attractive to mechanically stimulate more complex 3D multicellular tissues and organoids. The precision of optical stimulation could, however, be used to impart differential force modulation in larger cellular constructs, e.g., stimulating a specific region of an organoid, which could help understand how forces are communicated between stimulated and unstimulated cells.

## NPs for Mechanical Stress Modulation

While current tissue and matrix mechanics modulation approaches provide means to globally impose forces *in vitro*, in most cases such forces lack the heterogeneity in magnitude and direction that is more familiar to *in vivo* scenarios. In contrast, NPs dispersed in a matrix impart mechanical changes within their immediate vicinity, and since their distribution can be controlled, such alterations can be local or global, as well as on demand. Importantly, due to their capacity to be internalized by cells or to be tethered to targeted cells, they can directly impose forces in tissues on a cellular level (Figure 1B).

NPs come in many different shapes, sizes and materials, with a common feature that their characteristic length is less than 100 nm. NP types include quantum dots (Michalet et al., 2005), spherical particles (Pankhurst et al., 2003), rod and tube shaped particles (Wong et al., 1997), 2D sheets (Saliev et al., 2019; Zhu et al., 2019), and 3D superlattices (Ji et al., 2019). In the context of biological applications, NPs have been hailed for their ability to target cells of interest, shown most strikingly in targeting cancer cells to deliver drugs (Breunig et al., 2008), improving transfections (Sokolova et al., 2019), silencing genes (Frede et al., 2016; Morgan et al., 2019), or even employing material properties to destroy target cells (Sanhaji et al., 2019), all in an effort to combat disease. NPs are now increasingly being used in theranostic applications, an emerging field in medicine that combines targeted therapies with disease monitoring (Scialabba et al., 2019). The importance of these applications has prompted the development of new platforms to screen for delivery efficiency in tissues, and organoids are playing an increasingly important role here as biomimetic disease models (Davoudi et al., 2018; Leite et al., 2019). The functions that NPs can play in a biological setting are constantly being expanded, with mechanical stimulation of matrices and tissues figuring prominently as an important new application area.

## Local Modulation of Matrix Mechanics

In a NP-free matrix, polymer chains crosslink through bridging sidechains. The number of bridges formed during polymerization can determine the elasticity of the material, where relatively few crosslinks allow for freedom of movement and result in more elastic or viscoelastic bulk material behavior, while more crosslinks result in a more rigid material. When NPs are dispersed in a matrix, they can enhance or degrade its mechanical integrity on a local scale. If the size of NPs added to such a polymeric matrix is sufficiently small to fit within the interchain gaps while avoiding crosslink disruption, the more rigid material properties of the NPs relative to the polymers leads to a reduction in deformation and an increase in local stiffness. The incorporation of large NPs, or of NPs that disrupt the crosslinking process, has an opposite effect, creating locally suboptimal crosslinks and leading to a reduction in the local stiffness of the material. When considering the matrix as a whole, the accumulation of local nano-variations in mechanical properties can translate to bulk stiffness enhancement (Jestin et al., 2008) or hindrance (Abdel Fattah et al., 2016). One of the main uses of NPs in tissue engineering applications thus far has been to generate composite materials with enhanced properties, often as reinforcement material to strengthen the host matrix (Compton and Lewis, 2014). For example, when added to type I collagen, polyvinyl pyrrolidone-coated titanium oxide NPs have been shown to strengthen the resultant 3D scaffold and promote skin growth compared to NP-free matrices (Li N. et al., 2016). Similarly, fibrous materials whose properties would be ideal for growth of hard tissues such as bone but suffer from mechanical weakness have benefitted from NP reinforcement; for example NP-reinforced silk fibroin scaffolds successfully supported osteoblast cell culture (Kim et al., 2014). 3D bioprinting technologies have also benefited from the inclusion of nano filler materials, such as gelatin NPs in thixotropic collagen and hyaluronic acid bioinks for the culture of Hep2G organoids (Clark et al., 2019).

## Active Manipulation of Matrix Mechanics

**FIGURE 2:**
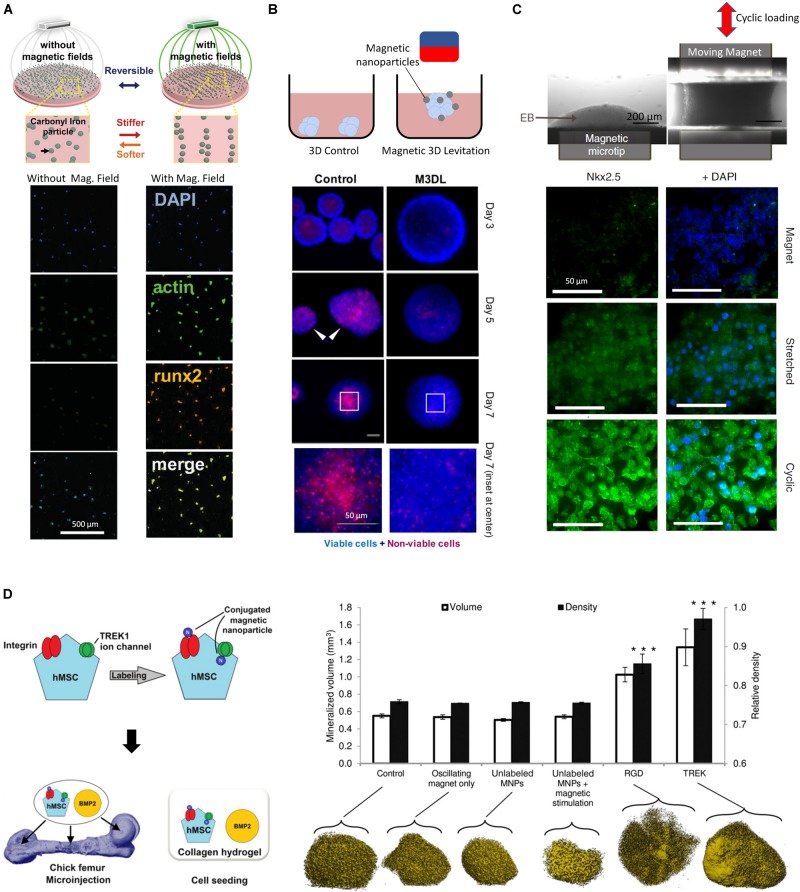
**(A)** Dynamic and reversible modulation of matrix stiffness using magnetic NPs and external magnetic fields influence the activity of MSCs (adapted from Abdeen et al., 2016). A magnetic field allows for the directed assembly of magnetic particles into chains, stiffening the mechanical properties of the microenvironment, resulting in MSCs with increased area. **(B)** Internalized magnetic NPs in salivary gland-derived cells accelerate spheroid formation by an external magnetic field, yielding faster and more reproducible spheroids with lower necrosis (Ferreira et al., 2019). **(C)** Internalized magnetic NPs allow remote manipulation of embryoid bodies and direct the differentiation of embryonic stem cells toward a mesodermal cardiac identity (adapted from Du et al., 2017). A cyclic magnetic field results in highest expression of mesoderm marker Nkx2.5 compared to a static magnetic field. **(D)** Magnetic NPs conduct remote mechanotransduction by (targeting mechanosensitive channels and receptors on cellular membranes (Henstock et al., 2014). Functionalized magnetic NPs designed to target the mechanosensitive TREK1 ion channel and integrins by RGD coating on hMSCs were injected in an *ex vivo* chick fetal femur. The combination of stimulation with BMP2 and oscillating magnetic field increased mineralization volume and density in targeted cells. *** indicates *p* < 0.001.)

While the addition of NPs in matrices can modulate their properties, NPs that are responsive to exogenous fields present unique advantages due to their ability to create anisotropic changes in the mechanical field in 3D (Jestin et al., 2008), and to create heterogeneous force distributions in the host matrix (Kokkinis et al., 2015; Abdalla et al., 2016; Abdel Fattah et al., 2016), allowing organoids and tissues to experience a spatio-temporally varying mechanical environment. Indeed, engineered NPs allows for continuous modulation of the mechanical stresses imposed on tissues through the control of the external activation field, which can be electric, optical, acoustic or magnetic. Magnetic fields in particular can be engineered with high precision within the dimensions of the culture environment using permanent magnets and high gradient magnetic fields. For example, carbonyl iron particles embedded into a polyacrylamide composite hydrogel have shown promise in reversibly modulating substrate stiffness from 0.1 to 90 KPa (Abdeen et al., 2016). When exposed to a magnetic field, the particles within the substrate align in chains, which present less deformable zones in the matrix and thus cause the substrate to become stiffer. The stiffness is reverted when the field is discontinued since the particles cannot maintain their alignment and diffuse to a homogenous distribution through Brownian motion. This physical reversibility is an important aspect of mechanically responsive matrices (Sapir-lekhovitser et al., 2016), as it allows for cyclic and variable activation with minimum material plasticity, leading to biological effects illustrated by an increase in cultured MSC spread area (Figure 2A). An increase in cell surface area here indicated that cells were developing higher tractions, which was accompanied by an increase in the expression of the osteogenic fate marker Runx2. In a separate study, magnetic NPs were dispersed in a PEG hydrogel to render it magnetic (Filippi et al., 2019). When subjected to an external magnetic field, cells from the stromal vasculature fraction of human adipose tissue were activated by movement of the NPs through the gel. Magnetically activated cells exhibited more metabolic activity, upregulated endothelial, pericytic and perivasculature markers, and activated pathways involved in mechanotransduction such as ERK and MAPK. These studies highlight the potential of magnetically responsive matrices to regulate cell function and fate specification; while matrices embedded with field-responsive NPs are not yet widely explored, they provide an exciting technology platform for exploring questions in mechanobiology.

## Generating Forces From Within: Cellular Internalization of NPs

While mechanical modulations external to cellular bodies can be achieved by embedding NPs within 3D ECMs, in order to exert a force directly within a tissue NPs must be internalized inside target cells. Synthesized NPs are not inherently biocompatible, therefore, in order to use them in biological applications, their surface chemistry must be modified in order to limit detrimental interactions with cells. In general, NPs enter cells through endocytosis and may accumulate to toxic levels or degrade within the cell due to oxidation, releasing harmful bioactive components leading to apoptosis or necrosis (Schweiger et al., 2012). NPs can also interfere with cytoskeletal rearrangement within the target cell, which can disregulate key cellular process such as cell division or migration (Bouissou et al., 2004; Mcbeath et al., 2004; Müller et al., 2013). For example, differentiating neural stem cells exposed to high levels of silver NPs exhibit disrupted β-catenin signaling, an important modulator of the cytoskeleton, via the emergence of inclusions in F-actin filaments (Cooper et al., 2019). These disruptions have been shown to lead to morphological changes such as reduction of neurite length, highlighting the vulnerability of neural cells to NP accumulation in the brain.

To overcome such cytotoxic effects, NPs can be rendered biocompatible through feature selection or surface functionalization, which allow for internalization routes that do not adversely disrupt the cell (Hsiao et al., 2019). Several types of internalization processes may be involved depending on NP size, shape, charge, and coating. NP size is often considered the main factor in determining whether the internalization mechanism occurs via phagocytosis or pinocytosis. Phagocytosis, due to the size of phagosomes, occurs for particles that are larger than ∼250 nm, while pinocytosis, responsible for the uptake of fluid and solutes, can internalize NPs ranging below ∼100 nm (Panariti et al., 2012). NP shape can also affect cellular uptake: spherical NPs, for example, have an internalization efficiency which is several orders of magnitude higher than nanorods of comparable feature length (Chithrani and Chan, 2007). This may be attributed to the longer time and higher energy needed to internalize asymmetrically shaped particles compared to their spherical counterparts.

In addition to NP shape, the negative charge of the plasma membrane makes it difficult for neutral or negatively charged particles to be internalized. To overcome this problem, NP surface charge modification may be necessary. Cationic liposome-coated magnetite NPs can be easily internalized by cells because they adopt a positive charge, which allows them to be attracted to the negatively charged plasma membrane, and to subsequently be internalized by the cell (Ito et al., 2005). A study of chitosan-based NPs of various surface charges demonstrated this principle in a systematic study where neutral particles had the lowest internalization efficiency across several cell types, followed by those with a negative charge, with optimal cellular internalization with positively charged NPs (Yue et al., 2011). However, higher uptake of positively charged NPs does not necessarily mean better NP performance, since such particles also tend to cause more extensive cytotoxic effects by inducing reactive oxygen species (ROS) production and apoptosis (Feng et al., 2018). This effect can be remedied through additional coatings of the NPs, which increase the overall NP size, but are designed to reduce the positive charge along with cytotoxic effects (Hoskins et al., 2012).

## Using NPs to Build 3D Spheroids and Shape Complex Geometries

Once NPs are internalized, they can be activated remotely using a variety of fields, including optically by infrared radiation (Liu et al., 2016). Optical tweezer approaches, for example, have been extensively used to exert forces or trap cells, often using external particles tethered to cellular membranes as conduits to convert optical power gradients into mechanical forces (Gao et al., 2017). As with the use of microbeads, NP-based optical tweezer techniques work well in planar 2D configurations, however in larger three-dimensional tissues, the choice of the activation field becomes limited, since such fields must penetrate the cells to reach the NPs. Magnetic fields in particular are able to penetrate biological materials with virtually no field disruption while remaining benign to tissues, which makes magnetic NPs a promising approach for remotely imparting mechanical forces directly on tissues.

Providing magnetic NPs for internalization lends cells magnetic responsiveness which can help form geometrical structures by guidance from an external magnetic field. Magnetic force generation in tissues can therefore have other applications beyond mechanotransduction, such as cell manipulation to create 3D cellular constructs. In addition to guiding cells to a specific geometry, magnetic levitation has been used to create 3D cellular spheroids (Souza et al., 2010). Magnetic fields have been used to create 3D constructs from cell cultures using paramagnetic salts (Abdel Fattah et al., 2018), however this technique provides a weak global body force which limits the effective range within which cells can be manipulated. In addition, the presence of salts means that culture durations must be limited to less than 24 hrs. In contrast, the use of magnetic NPs was shown to increase forces and effective manipulation range, which enabled the formation of highly reproducible spheroids. By applying a magnetic field, magnetized salivary gland-derived cells were guided to the air-media interface where they agglomerated to form spheroids which eventually differentiated into gland-like organoids (Figure 2B) (Ferreira et al., 2019). This approach provided rapid cellular assembly while foregoing the need for scaffold materials, rendering this a promising advance towards salivary gland repair. Similarly, spinal cord spheroids could be magnetically assembled with high reproducibility by magnetic NPs adhering to the membranes of dissociated primary spinal cord cells (Bowser and Moore, 2019), forming neurites upon seeding in a hydrogel matrix.

In order to create more complex geometries, experimental configurations utilizing shaped magnetic fields can be employed. In one example, urothelial, endothelial, smooth muscle, and fibroblasts cells with internalized magnetic NPs were guided into a tubular 3D multilayered cellular construct with a 5 mm lumen using a cylindrical magnet (Ito et al., 2005). This technique, also known as magnetophoresis, allows for versatile control over geometrical assembly, as demonstrated by the guidance of magnetized C2C12 myoblasts into multilayered myotube rings, thereby promoting differentiation into highly aligned skeletal muscle tissue (Yamamoto et al., 2009).

Going from bulk cell activation to targeting and mechanically stimulating specific cell populations within a tissue would represent an important advance in our ability to recapitulate the complex and heterogeneous force fields experienced *in vivo*. This level of control over spatial positioning and mechanical stimulation with high spatial resolution has been achieved in a proof-of-principle study using magnetic NPs internalized by HeLa cells plated on a magnetic array, where forces could be imposed at a resolution of less than a cell diameter (Tseng et al., 2012).

## Combining Multicellular Shaping and Timed Mechanical Stimulation

In addition to creating 3D cellular constructs and designed geometries at the initiation of an experiment, magnetic NPs can provide mechanical stimulation at later time points. This two-step process was demonstrated with the initial aggregation of embryonic stem cells (ESCs) into embryoid bodies after uptake of iron oxide NPs by an external magnetic field (Figure 2C), followed by the re-purposing of the magnetic NPs as mechanical stimulators imparting oscillatory strains, actuated through a combination of magnetic and magneto-mechanical activation (Du et al., 2017).

Since the magnitude of exerted forces is a function of total internalized NPs, assessing their uptake is important. While chemical assays such as the potassium thiocyanate method are commonly used to assess internalization (Ito et al., 2005), other quantitative approaches have been developed. For example, pulling free-floating magnetized cells by a magnetic field through a medium and monitoring their velocities can provide a quantitative inference of the mass of the internalized NPs by balancing the forces of fluid drag under Stokes condition and that of the magnetic force. Thus, when NP internalization is sufficient, cyclic magneto-mechanical stimulation can be sufficient to direct the ESC differentiation to mesodermal cardiac fate (Du et al., 2017). Similarly, chondrogenic fate specification and collagen production can be enhanced magnetized hMSCs are subjected to cycling static and rotating external magnetic fields (Son et al., 2015). Notably, the magnetic NPs used in this study were produced by magnetospirillum sp. AMB-1 bacteria, highlighting the promise of biologically derived magnetic NPs, which are thought to be more readily internalized due to residual lipid layers surrounding the NPs.

## Achieving Cell Selectivity and Cell Targeting With NPs

In addition to aiding in internalization, the surface coating of NPs can be bioengineered to achieve enhanced targeting accuracy of particular cell types by mimicking specific ligands and exploiting cell-specific receptor-based endocytosis (Breunig et al., 2008; Foroozandeh and Aziz, 2018). These bioconjugation processes are often designed in conjunction with surface charge modification, as both must be optimized to avoid undesirable NP aggregation. To achieve high cell selectivity NP coatings can be designed to promote targeted interactions with the cellular membrane, becoming robustly tethered only to those cells that express receptors compatible with the coating (Hughes et al., 2007). For example, when hMSCs were treated with magnetic NPs targeting the mechanosensitive TREK1 ion channel, then injected into an *ex vivo* chick fetal femur and stimulated by an externally oscillating magnetic field, the magnetized NPs produced a 4pN force upon the receptor, initiating a remote mechanotransductive effect and leading stimulated cells to exhibit increased mineralization (Henstock et al., 2014) (Figure 2D). This approach was implemented in an *in vivo* pre-clinical ovine bone injury model and demonstrated the possibility of remote force induction in a large animal model to promote bone healing (Markides et al., 2018). Similar results were obtained when magnetic NPs were functionalized to target Frizzled, the receptor for Wnt (Rotherham et al., 2018), on magnetically stimulated hMSCs, suggesting that this is a versatile strategy for applications in osteogenesis. This approach is transferrable to other lineages, requiring mainly the modification of the magnetic NP functionalization to target different receptors of interest. For instance, when targeting the activin receptor type IIA on human adipose stem cells, a varying magnetic field helped activate the receptor and promoted tenogenic differentiation, providing a promising result for tendon repair applications (Gonçalves et al., 2018). Interestingly, magnetic NPs can also impose such high forces that cell death can result – in some case this may be valuable, for example to mechanically disrupt and destroy cancer cells (Shen et al., 2017) such as malignant glioma cells in- tumor-bearing mice, which could be destroyed by the application of a rotating magnetic field to internalized disk shaped permalloy magnetic NPs (Cheng et al., 2016). In the context of organoid bioengineering, targeting of specific cells through surface markers for mechanical activation could allow for selective activation of cells of specific fates within a heterogenous organoid.

## Outlook

The context-specific manner in which cells in tissues sense, react and in turn generate mechanical force has become an active area of research. In order to explore the relationship between mechanical forces and fate specification and morphogenetic outcomes, the development of *in vitro* multicellular model systems with local and directed force generation is critical. NPs provide unique and versatile features which enable such investigations, including control over spatial resolution, targetability as well as remote actuation which, alone or in combination with aECMs, allow for highly specific organoid manipulation. Magnetic NPs in particular are likely to see the greatest application and development in the field, as they are relatively benign and need little modification for efficient uptake by most cells. Additionally, they are also easily synthesized using straightforward benchtop fabrication techniques with simple chemistries such as co-precipitation. Magnetic NPs dispersed in a matrix can provide active mechanical stimulation in any direction, rendering them more versatile than conventional methods such as stretching devices, which are generally limited to strains along predefined orientations. While magnetic NPs are useful tools for matrix as well as tissue stimulation, magnetization of cells through NP internalization is likely to see more applications due to the ability of internalized magnetic NPs to create spheroids and organoids by magnetic guiding, and to then generate internal magnetic forces in host matrices.

Assessing the changes in mechanical state upon stimulation is critical in validating the relationship between applied force and biological outcomes. The mechanical dynamics in matrices can be interrogated using techniques such as traction force microscopy (TFM) (Jorge-Peñas et al., 2017) while those within tissues can be assessed by force inference (Veldhuis et al., 2017), fluorescence resonance energy transfer (FRET) biosensors (Grashoff et al., 2010), or dispersible and deformable hydrogel microparticles embedded within the tissue (Lee et al., 2019). Such force interrogation methods are, in principle, fully compatible with NP-mediated mechanical stimulation of the matrix or tissue. In particular, bead tracking techniques and TFM, which assess external matrix mechanics, can be beneficial for NP-mediated matrix mechanical modulation. In contrast, FRET-based sensors convey information about tissue deformations and stresses arising from direct tissue stimulation through internalized NPs.

Because NPs can be stimulated by external fields which can be engineered with high precision, such as light or magnetism, a dispersion of NPs can be homogeneous (global) while the stimulating field is highly localized. Such effects can help introduce anisotropic manipulation of organoid microenvironments. In contrast, mixing magnetized cells with non-magnetized cells in specific ratios can create cellular constructs which have specific subpopulations that are magnetically responsive. In this case a relatively local magnetic response could be obtained within the organoid using a global magnetic field, either by permanent magnets or electromagnets. Since NPs can be easily modified, they can serve multiple mechanical and biochemical purposes and can act as mechanotransducers via one field, as well as morphogen carriers released by degradation or in response to a secondary field.

As NPs are generally not in current use in organoid and tissue engineering labs, their adoption would also add a few hurdles. Since uptake and cytotoxic effects are likely to vary between different cell types and also between differentiation protocols, these will have to be tested for individual model systems. In addition, external field activation would require the acquisition and implementation of new instruments and expertise, with consequent additional costs and complexity to experimental planning. Finally, as with current organoid culture and bioengineering approaches, reproducibility of these new and unconventional approaches will require extensive characterization to achieve robust and reproducible outcomes. Despite these current challenges, the studies we have reviewed here have demonstrated that NPs can be powerful new tools to modulate the mechanical properties of the microenvironment, and it is expected that in time, simple NP uptake, cell targeting and dispersal protocols are likely to emerge and pave the way to wider use in organoid and tissue engineering applications.

## Author Contributions

AA and AR wrote and edited the manuscript.

## Conflict of Interest

The authors declare that the research was conducted in the absence of any commercial or financial relationships that could be construed as a potential conflict of interest.
